# Ischemic Colitis in Sickle Cell Disease: A Case Report of a Diagnostic Challenge

**DOI:** 10.1155/2018/2358091

**Published:** 2018-01-24

**Authors:** Kyle Geary, Jacob Kibrit

**Affiliations:** Department of Internal Medicine, University of Illinois at Chicago, Chicago, IL 60612, USA

## Abstract

Microvascular occlusion serves as the underlying mechanism for the multitude of clinical manifestations of sickle cell disease, one of the most prevalent hemoglobinopathies worldwide. Recurrent painful episodes are the hallmark of this condition. Abdominal pain attributed to an acute painful episode can be indistinguishable from a separate and/or more serious intra-abdominal disease process, representing a significant diagnostic dilemma for clinicians. Here we present a rare case of ischemic colitis due to vascular occlusion in a 28-year-old man with sickle cell disease.

## 1. Introduction

 Hemoglobin S (HbS) disease is an autosomal recessive disorder related to a single nucleotide substitution (glutamic acid to valine at position 6) in the beta-globin chains, which combine with alpha-globin subunits to form hemoglobin S (HbS). The deoxygenated HbS forms long polymers, which ultimately result in the misshapen, “sickle” cell shape. This elicits a complex pathway of erythrocyte dehydration and abnormal erythrocyte/leukocyte-endothelial adhesion, triggering an inflammatory cascade that results in the classic postcapillary vasoocclusive complications associated with the disease [1]. A hallmark of the disease is recurrent vasoocclusive painful episodes. We present a rare case of ischemic colitis due to microvascular occlusion in a patient with hemoglobin S (HbS) disease.

## 2. Case Report

**Figure 1:**
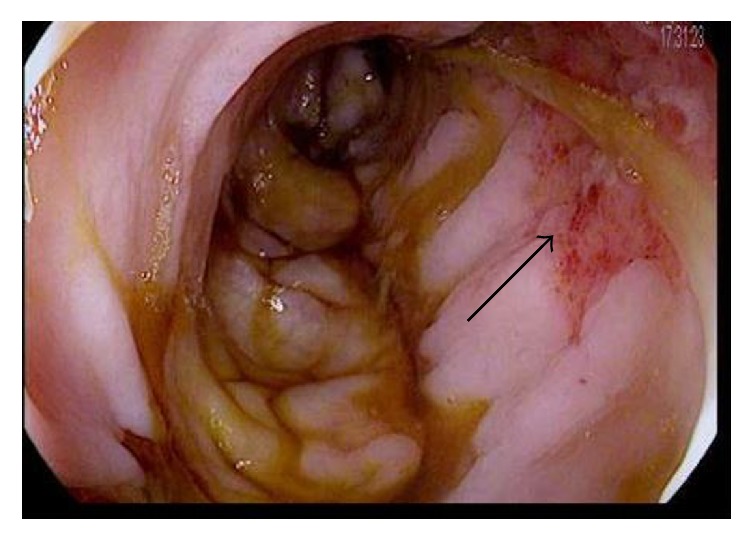
Endoscopic image of the sigmoid colon with areas of mucosal hemorrhage and inflammation.

**Figure 2:**
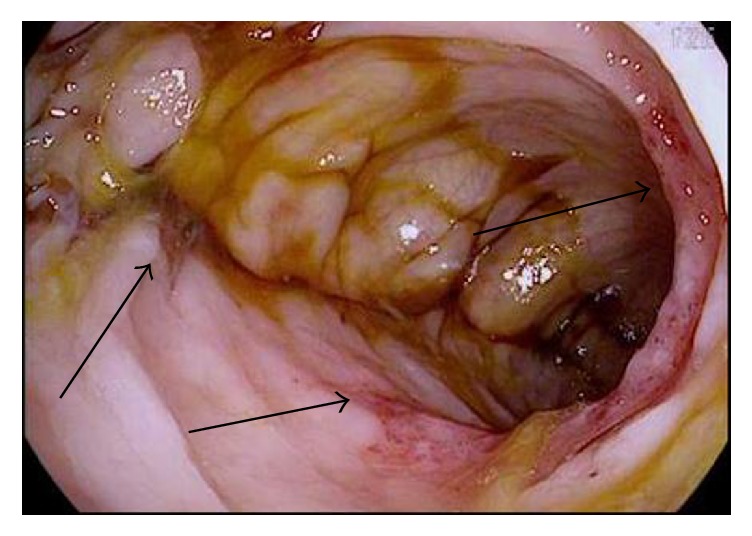
Endoscopic image of the sigmoid colon with areas of mucosal hemorrhage, mucosal friability, and inflammation.

**Figure 3:**
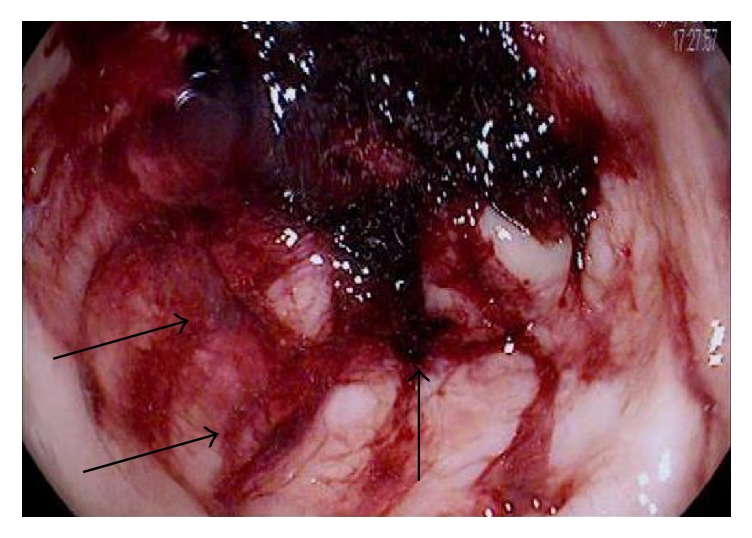
Endoscopic image of the rectum with a significant amount of mucosal hemorrhage along with large ulcerations covered in hematin.

**Figure 4:**
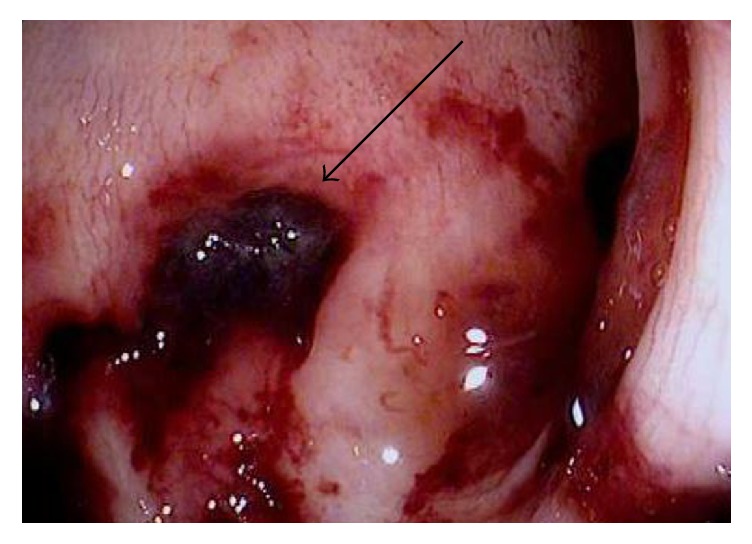
Endoscopic image of the descending colon with mucosal hemorrhage and a large actively bleeding ulceration.

A 28-year-old gentleman with a history of sickle cell disease (HbS) complicated by recurrent acute painful episodes, acute chest syndrome, and iron overload secondary to prophylactic chronic exchange transfusion therapy presented to the emergency department with abdominal pain, back pain, and diarrhea. The patient was previously admitted to the hospital twice within the month for similar symptoms with an unrevealing work-up, and his abdominal pain and diarrhea were thought secondary to adverse effects from the deferasirox he was receiving for chronic iron overload. On initial presentation of this admission, the patient was afebrile and hemodynamically stable but in moderate discomfort. His exam was significant for normal bowel sounds and a soft, nondistended, diffusely tender abdomen, particularly in the left lower quadrant, without guarding or rebound tenderness. Initial laboratory studies were significant for a white blood cell count of 22,500 with 90% neutrophils; otherwise, additional blood count and chemistry parameters were unchanged from the patient's baseline studies. Intravenous fluid hydration and patient-controlled analgesia (PCA) was initiated. On hospital day 2, the patient developed two episodes of bright red blood per rectum, a first reported occurrence per the patient; rectal exam was unrevealing. The patient was made “nothing by mouth” and the Gastrointestinal (GI) medicine team was consulted. A CT (computed tomography) of the abdomen and pelvis with oral and intravenous contrast was performed and demonstrated small focal diverticula at the splenic flexure. On hospital day 3, a flexible sigmoidoscopy was performed which revealed friable and erythematous mucosa, along with a decreased vascular pattern and ulcerated areas from the sigmoid to splenic flexure. Figures 1–4 illustrate the endoscopic findings from the flexible sigmoidoscopy. Biopsies of the descending colon demonstrated focal acute inflammation, mucosal hemorrhage, fibrin, and distorted crypts, consistent with ischemia. Based on the endoscopic and biopsy findings, the diagnosis of ischemic colitis was made. On hospital day 5, the patient received an exchange transfusion with successful reduction of serum HbS (hemoglobin S) from 42% to 12%. The patient's hematochezia and abdominal pain resolved on hospital day 6 and he was discharged with plans to increase exchange transfusion frequency from bimonthly to monthly, along with continuation of his previously discontinued deferasirox. The patient was subsequently admitted to the hospital for another vasoocclusive episode several weeks later but denied any recurrence of abdominal pain or hematochezia at that time.

## 3. Discussion

Abdominal pain in patients with HbS disease can represent a diagnostic challenge for clinicians. The disease can often result in generalized and diffuse pain, which can be exacerbated by adverse effects of therapies such as constipation from opioids or abdominal pain from deferasirox. The finding of ischemic colitis as an etiology of abdominal pain in a patient with HbS disease is relatively uncommon; however, clinicians who treat this population must be cognizant of this diagnosis. Ischemic colitis is classically a “supply and demand” disease of the elderly; the inadequate blood supply may be secondary to hemodynamic instability (“shock”) or thromboembolic events to the colonic blood supply secondary to conditions such as atrial fibrillation, cardiac valve disease, acute MI, recent cardiac surgery, or mechanical obstruction (from mass or tumor).

In the younger population, ischemic colitis can be seen in patients with systemic vasculitides, excessive stimulant (cocaine, amphetamine) usage, long-distance runners, and those with underlying inheritable hypercoagulable disorders (most commonly factor V Leiden mutation) [2]. The colon is supplied by two large feeder arteries, the superior and inferior mesenteric arteries. The superior mesenteric artery supplies from the 2nd portion of the duodenum to the splenic flexure, joining with the inferior mesenteric artery (which supplies from the splenic flexure to the upper rectum) at an anastomosis called the marginal artery of Drummond. The marginal artery of Drummond is located at the splenic flexure and notoriously has few to absent collateral circulation; this absence of collaterals means the splenic flexure is at high risk of ischemia in episodes of hemodynamic instability or thromboembolic events to one of the mesenteric arteries [2]. Our patient had findings suggestive of ischemia from the sigmoid and descending colon down to the upper rectum, suggesting microvascular occlusion at the level of the inferior mesenteric system.

Regardless of etiology, symptoms generally include abdominal pain, diarrhea, and hematochezia. If left untreated, bowel necrosis can lead to a septic picture, including hypotension, tachycardia, altered mental status, fever, and laboratory findings such as lactic acidosis, leukocytosis, or organ dysfunction. Diagnosis often includes abdominal imaging with intravenous contrast, where CT findings could include bowel wall thickening, edema, and inflammation [3]. Definitive endoscopic findings of friable mucosa, mucosal hemorrhage, and inflammatory changes are diagnostic. Early intervention includes intravenous fluids and close observation for systemic signs of toxicity, for which antibiotic and surgical therapy should be considered.

In patients with HbS disease suspected of having ischemic colitis, defining appropriate management strategies is challenging given the paucity of literature available. In the case reports published, varying combinations of supportive care (bowel rest, intravenous fluids), broad spectrum antibiotics, exchange transfusion, and surgical management have all been implemented. Despite these interventions, several case reports describe significant complications, including bowel perforation and shock resulting in death [4, 5]. It is important that clinicians have a high index of suspicion for this disease and consider early abdominal imaging and thorough laboratory and physical exam investigation.

## References

[1] Stuart M. J., Nagel R. L. (2004). Sickle-cell disease. *The Lancet*.

[2] Theodoropoulou A. (2008). Ischemic colitis: clinical practice in diagnosis and treatment. *World Journal of Gastroenterology*.

[3] Gardner C. S., Jaffe T. A. (2015). CT of gastrointestinal vasoocclusive crisis complicating sickle cell disease. *American Journal of Roentgenology*.

[4] Abadin S. S., Salazar M. R., Zhu R. Y., Connolly M. M., Podbielski F. J. (2009). Small bowel ischemia in a sickle cell patient. *Case Reports in Gastroenterology*.

[5] Karim A., Ahmed S., Rossoff L. J., Siddiqui R., Fuchs A., Multz A. S. (2002). Fulminant ischaemic colitis with atypical clinical features complicating sickle cell disease. *Postgraduate Medical Journal*.

